# Toward Prospective Prediction of Pharmacokinetics in OATP1B1 Genetic Variant Populations

**DOI:** 10.1038/psp.2014.50

**Published:** 2014-12-10

**Authors:** R Li, H A Barton, T S Maurer

**Affiliations:** 1 Systems Modeling and Simulation, Department of Pharmacokinetics, Dynamics, and Metabolism, Pfizer Worldwide R&D, Cambridge, Massachusetts, USA; 2 Department of Pharmacokinetics, Dynamics, and Metabolism, Pfizer Worldwide R&D, Groton, Connecticut, USA

## Abstract

Physiologically based pharmacokinetic (PBPK) models are increasingly being used to provide human pharmacokinetic (PK) predictions for organic anion-transporting polypeptide (OATP) substrates based on *in vitro* assay data. As a natural extension in the application of these models, in this study, we incorporated *in vitro* information of three major OATP1B1 genetic variants into a previously reported PBPK model to predict the impact of OATP1B1 polymorphisms on human PK. Using pravastatin and rosuvastatin as examples, we showed that the predicted plasma concentration–time profiles in groups carrying different OATP1B1 genetic variants reasonably matched the clinical observations from multiple studies. This modeling and simulation approach may aid decision making in early pharmaceutical research and development as well as patient-specific dose adjustment in clinical practice.

Membrane transporters play a significant role in the pharmacokinetic (PK) profiles of many drugs,^1,2^ to which functional genetic variations in transporters can contribute. The organic anion-transporting polypeptide 1B1 (OATP1B1), encoded by the gene *SLCO1B1*, is an uptake transporter expressed on the basolateral membrane of human hepatocytes. OATP1B1 has a broad substrate specificity and is important in both systemic and liver exposure of many drugs.^3^ OATP1B1 genetic variants with decreased activity may increase systemic exposure, and hence the chance of dose/concentration related adverse drug reactions. For example, the incidence of severe myopathy with statin (OATP1B1 substrates) therapy is related to dose.^4^ On the other hand, genetic variants with increased activity may decrease the plasma concentration to subtherapeutic levels. Additionally, the impact of OATP1B1 polymorphisms on liver exposure, associated pharmacodynamic effect of liver-targeting compounds, and potential toxicity of nonliver-targeting compounds have not been well characterized and deserve attention.

Multiple *SLCO1B1* single nucleotide polymorphisms (SNPs) have been identified, among which there are two that are relatively common and widely studied, i.e., c.521T>C in exon 5 and c.388A>G in exon 4 (where c. represents coding DNA sequence, the number refers to the first nucleotide affected, and “>” indicates a substitution at DNA level). The two SNPs lead to four haplotypes, known as *1a (wild-type, c.388A and c.521T), *1b (c.388G and c.521T), *5 (c.388A and c.521C), and *15 (c.388G and c.521C).^3^ Adding to the complexity, individuals can be homozygous or heterozygous for these haplotypes. Compared with *1a, the haplotypes *5 and *15 usually have decreased uptake rate,^3^ while *1b may increase the uptake rate due to increased OATP1B1 expression.^5^ Although the prevalence and functional implications of other *SLCO1B1* SNPs lead to different haplotypes (e.g., *14 (c.388G, c.521T and c.463A)), these have not been widely studied as the four haplotypes listed above.^3^

The aim of this study is to predict human PK of OATP1B1 substrates for carriers of *SLCO1B1* variants through physiologically based pharmacokinetic (PBPK) modeling. Such a model could aid decision making in early pharmaceutical research as well as patient-specific dose adjustment in clinical practice. Rosuvastatin and pravastatin are used in this study to demonstrate the prediction strategy, because there are multiple *in vitro* pharmacogenetic and *in vivo* genotyped pharmacokinetic datasets available for the two compounds.

## Results

### PBPK modeling of nongenotyped clinical data and estimation of OATP1B1 activity

**Figure 1:**
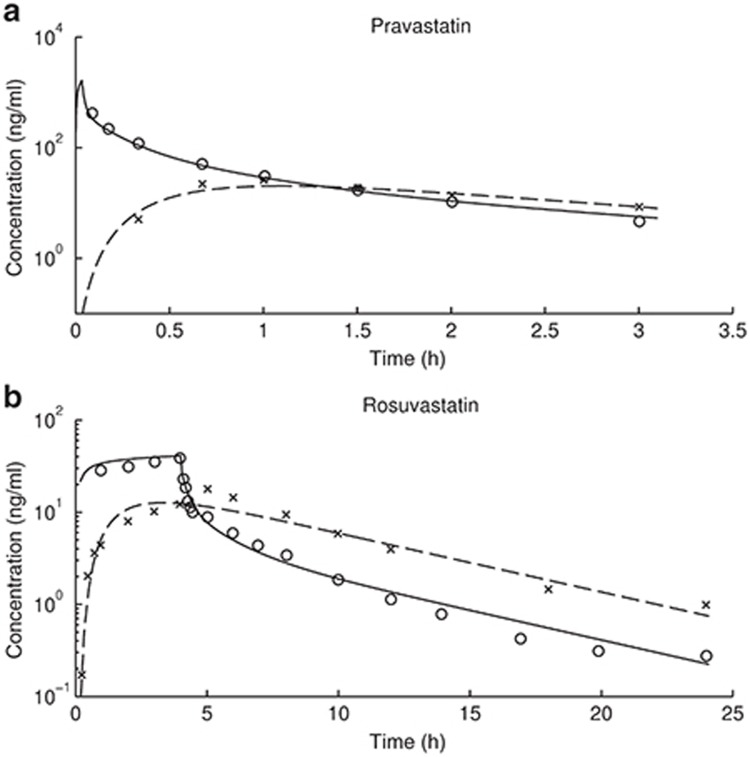
Observed and fitted human plasma time–concentration profiles of (**a**) pravastatin and (**b**) rosuvastatin. Circles and crossings represent observed profiles after IV infusion (9.9 mg for pravastatin, 8 mg for rosuvastatin) and oral dosing (19.2 mg for pravastatin, 40 mg for rosuvastatin), respectively.^6,7^ Solid and dashed lines represent simulations after IV infusion and oral dosing, respectively.

The nongenotyped clearance and absorption parameters including total hepatic active uptake clearance (*CL*_*act, tot*_), biliary clearance (*CL*_*bile*_), passive diffusion clearances in liver (*CL*_*pass, liver*_), absorption rate (*k*_*a*_), and the fraction of the dose absorbed from gastro-intestinal (GI) tract (*F*_*a*_*·F*_*g*_) are estimated by fitting nongenotyped mean intravenous infusion and oral dosing data.^6,7^ Model characterization of the data and the values of both fitted and predicted parameters generated in this study are indicated in **Figure 1** and **Table 1**. The model-derived values of *F*_*a*_*·F*_*g*_ for pravastatin and rosuvastatin (i.e., 0.46 and 0.50) are lower than what would be obtained via noncompartmental analysis (i.e., 0.52 and 0.72). This is likely due to an overestimation of *F*_*a*_*·F*_*g*_ via noncompartmental analysis in the presence of enterohepatic recirculation. Consistent with this, a smaller difference is observed with pravastatin due to the relatively greater contribution of renal (rather than biliary) clearance.

### Predictions of uptake clearance in OATP variants and PK in genotyped Caucasian and Japanese populations

**Figure 2:**
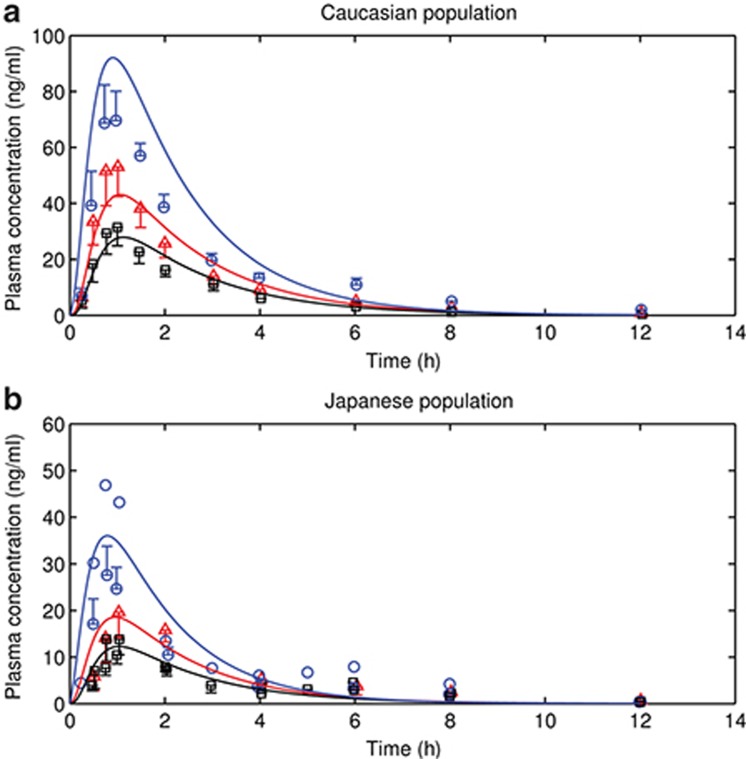
Observed and predicted human plasma time–concentration profiles of pravastatin (**a**) following 40 mg oral dosing in Caucasian population and (**b**) following 10 mg oral dosing in Japanese population. Red triangles and lines represent observed and predicted profiles of *1a group. Black squares and solid lines represent observed and predicted profiles of *1b group. Blue circles and lines represent observed and predicted profiles of *15 group. Error bars indicate observed standard deviations. The digitized observations are from the one study performed with Caucasian population, and two studies with Japanese population.^13,14,20^

The active uptake clearance of OATP1B1 *1a, *1b, and *15 (*CL*_act, *1a_, *CL*_act, *1b_, and *CL*_act, *15_) for the Caucasian population are calculated from *in vitro* data and nongenotyped active uptake clearance through OATP1B1 (*CL*_*act, OATP1B1*_) (**Table 1**) as described in the methods section, where *5 and *15 are treated as the same group due to relatively similar activity of the two variants.^8^ With calculated values of *CL*_act, *1a_, *CL*_act, *1b_, and *CL*_act, *15_, the model reasonably predicts observed human plasma pravastatin profiles of Caucasian *1a, *1b, and *15 groups (**Figure 2a**).

A previous publication indicates that there is an intrinsic ethnic variability in the activity of OATP1B1,^9^ where the ratio of Japanese/Caucasians is 0.584. Keeping all other parameters unchanged, predictions using this correction on *CL*_*act, tot*_ also match the observations in a previously published study on pravastatin pharmacokinetics in Japanese subjects (**Figure 2b**). A similar prediction is not done for rosuvastatin due to the lack of the genotyped rosuvastatin pharmacokinetic data in Japanese population.

**Figure 3:**
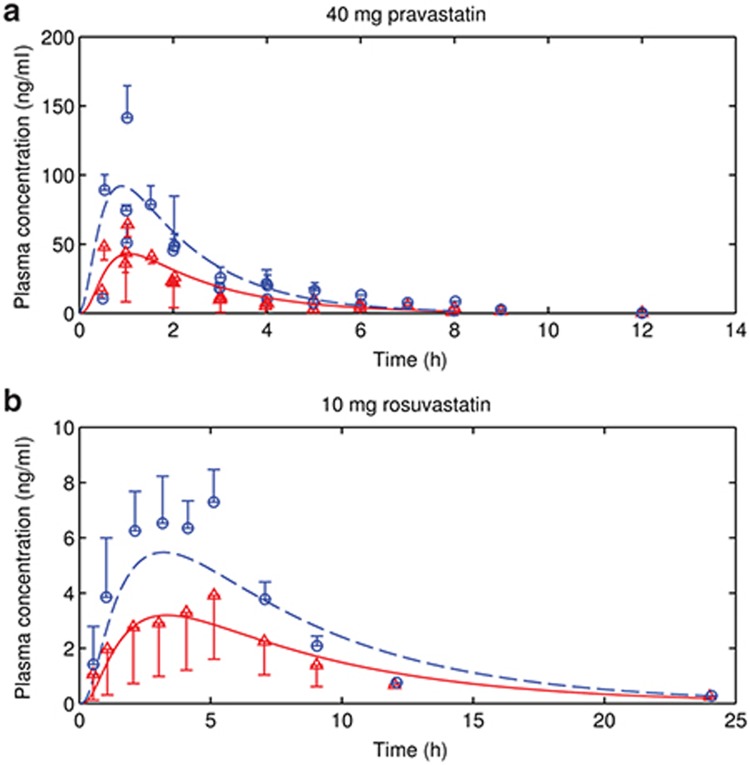
Observed and predicted human intravenous plasma time–concentration profiles of (**a**) pravastatin and (**b**) rosuvastatin in Caucasian population. Red triangles and blue circles represent observed c.521TT and c.521CC groups. Red solid lines and blue dashed lines represent predicted *1a and *15 groups, assuming c.521TT and c.521CC groups are equivalent to *1a and *15 groups, respectively. Error bars indicate observed standard deviations. The digitized observations are three studies for pravastatin, and one study for rosuvastatin.^15,16,17,18^

Pravastatin as well as rosuvastatin concentration–time profiles of c.521TT and c.521CC groups were also reasonably well predicted under the assumption that (in the absence of c388A>G information) these largely represent the *1a and *15 genotypes (**Figure 3**). The differences between the observations and predictions are within 70% of the observed values for plasma AUC, 75% of the observed values for *C*_max_, and 10% of the observed values for *t*_max_ (**Table 2**). Considering the relatively large intra- and interstudy variability (**Table 2**), the observed and predicted pharmacokinetics are reasonably close.

### Local sensitivity analysis

**Figure 4:**
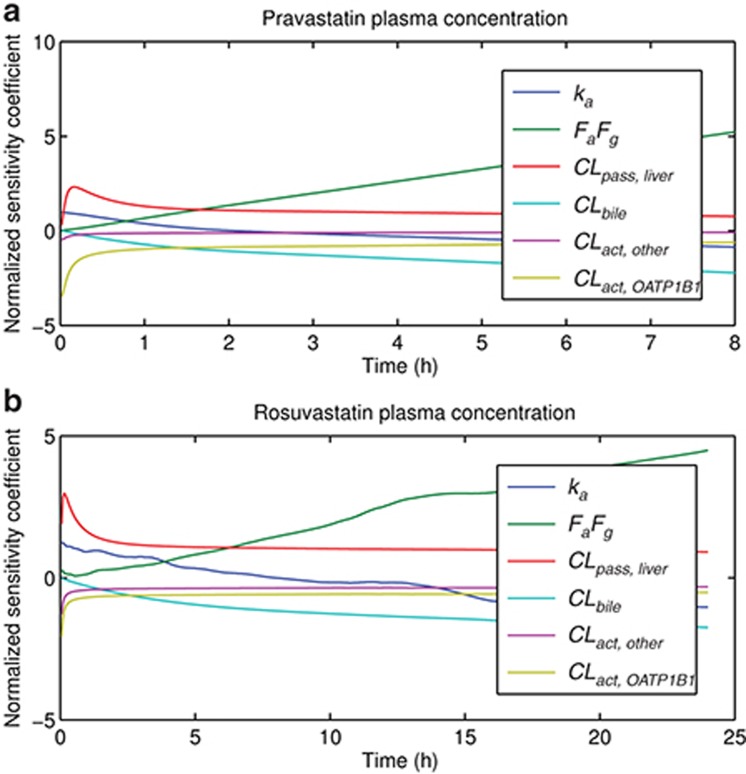
Time-dependent local sensitivity of compound specific parameters on plasma concentration of (**a**) pravastatin and (**b**) rosuvastatin. The local sensitivity analysis shown here is conducted with parameter values associated with OATP1B1 *1b group, however similar results are observed for *1a and *15 groups.

The sensitivities of plasma and liver concentration, and AUC_plasma_ up to 8 h for pravastatin and 24 h for rosuvastatin in OATP1B1 genotyped Caucasian population, were evaluated for compound specific parameters. The parameters with normalized sensitivity coefficients greater than 0.3 or less than −0.3 are reported. The plasma concentration is sensitive to unbound fraction in plasma (*f*_*u, p*_), blood to plasma ratio (*R*_*B/P*_), unbound fraction in liver tissue (*f*_*u, liver*_), renal clearance (*CL*_*renal*_), *CL*_*act, OATP1B1*_, active uptake clearance through non-OATP1B1 transporters (*CL*_*act, other*_), *CL*_*bile*_, *CL*_*pass, liver*_, *k*_*a*_, and *F*_*a*_*·F*_*g*_. The liver concentration is also sensitive to these parameters except for *R*_*B/P*_ and *CL*_*renal*_. Plasma concentration is more sensitive towards *CL*_*act, OATP1B1*_ than the liver concentration (data not shown), consistent with a previous PBPK study for pravastatin.^10^ In addition, the plasma concentration is sensitive to different parameters during different phases (**Figure 4**), consistent with previous observations.^11^ AUC_plasma_ is sensitive to *f*_*u, p*_, *CL*_*act, OATP1B1*_, *CL*_*act, other*_, *CL*_*bile*_, *CL*_*pass, liver*_, and *F*_*a*_*·F*_*g*_. In general, results are similar between pravastatin and rosuvastatin among *1a, *1b, and *15 groups, except that pravastatin AUC_plasma_ is not sensitive to *CL*_*act, other*_, and rosuvastatin AUC_plasma_ is not sensitive to *CL*_act, *15_.

Because the predicted *CL*_*act, tot*_ in genotyped populations are calculated using *in vitro* data, a local sensitivity analysis was performed on predicted *CL*_*act, tot*_ (in *1a, *15, and *1b populations) towards the parameters estimated in the *in vitro* assays (i.e., the ratio between *CL*_act, *1a_ and *CL*_act, *1b_, the ratio between *CL*_act, *1a_ and *CL*_act, *15_, and the fraction of *CL*_*act, tot*_ mediated by OATP1B1) (**Table 3**). In general, as the fraction of *CL*_*act, tot*_ mediated by OATP1B1 increases, the predicted *CL*_*act, tot*_ across populations is more sensitive to the ratio between *CL*_act, *1a_ and *CL*_act, *1b_ (or *CL*_act, *15_). When OATP1B1 mediates over 60% *CL*_*act, tot*_, the predicted *CL*_*act, tot*_ for all three genotyped populations is relatively sensitive to the ratio between *CL*_act, *1a_ and *CL*_act, *1b_ (i.e., the sensitivity coefficient is 0.3 or greater), while only *CL*_act, tot,*15_ is sensitive to the ratio between *CL*_act, *1a_ and *CL*_act, *15_ under most situations (i.e., only *CL*_act, tot,*15_ but not *CL*_act, tot,*1a_ or *CL*_act, tot,*1b_ has sensitivity coefficient larger than 0.1). Simulated plasma concentration–time profiles using the values of the ratio between *CL*_act, *1a_ and *CL*_act, *15_ reported from two *in vitro* studies (**Supplementary** **Figure S1**) are consistent with the sensitivity analysis results in **Table 3**, that only *CL*_act, tot,*15_ is sensitive to the ratio between *CL*_act, *1a_ and *CL*_act, *15_.

## Discussion

The effects of *SLCO1B1* polymorphisms on transporter activity for selected OATP substrates and human PK have been established in both *in vitro* and *in vivo* studies;^3^ however, a mechanistic model describing these behaviors and connecting *in vitro* discoveries with *in vivo* observations has not been developed previously. In this study, we incorporated *in vitro* OATP1B1 information for genetic variants into a previously published PBPK model for OATP substrates^12^ to predict PK profiles of variant carriers.

The uniqueness of this approach relies on the use of *in vitro* estimated fraction of OATB1B1 in total hepatic active uptake clearance and the ratio of uptake activities between variants. In combination with hepatic clearance and absorption estimated from average plasma concentrations of ungenotyped population, the proposed approach can reasonably predict plasma concentration–time profiles for genotyped groups. The prediction relies on the key assumptions that the contribution of OATP1B1 to total uptake and the effect of *SLCO1B1* polymorphisms on uptake activities are relatively consistent between *in vitro* and *in vivo* conditions, and only *CL*_*act, OATP1B1*_ changes without the need for adjustment of other parameters and the model structure.

Accounting for differences in OATP1B1 activity between Caucasians and Japanese,^9^ the model can also predict observations in Japanese pravastatin study^13,14^ (**Figure 2**). Application of the same approach to rosuvastatin pharmacokinetics determined in ungenotyped Japanese subjects suggests that the ethnic difference in OATP activity may be larger for this compound (0.3 rather than 0.584, data not shown). As such, further study is required to understand the genotype-dependence of rosuvastatin pharmacokinetics in Japanese and to determine the ethnic dependence of OATP uptake for this compound.

Given the available data, several assumptions are made to simplify the problem. We assume that the fraction of three OATP1B1 variants in nongenotyped Caucasian IV studies follows that in European population. We assume that heterozygotes have the same activity as the homozygotes if the pharmacokinetic study grouped heterozygotes and homozygotes together. This may lead to the misprediction of the pharmacokinetics, because heterozygous clearance may be different from homozygous clearance.^15^ We further assume that in **Figure 3** the c.521TT group is the same as the *1a group in the studies for which SNP c.388A>G or c.463C>A is not sequenced;^15,16,17,18^ the assumed *1a (c.521TT, c.388AA, and c.463CC) group may be confounded by *1b (c.521TT, c.388GG and c.463CC) and/or *14 (c.521TT, c.388GG, and c.463AA) genotypes. The *1b genotype is associated with the increased OATP1B1 expression level,^5^ while the *14 genotype may be associated with the reduction in the intrinsic OATP1B1 uptake rate.^19^ In addition, in **Figure 2** where data are digitized from the studies sequencing c.388A>G,^13,14,20^ *1b group could also be confounded by *14 carrier if c.463C>A was not genotyped. We further assumed that polymorphisms of other transporters only play minor roles in drug disposition and do not affect plasma PK, and no gene interactions between OATP1B1 and other transporters. The use of these assumptions is supported by the good agreement between predictions and observations in the two case examples.

*In vitro* assay data indicate *1b increases OATP1B1 expression by twofold,^5^ which is assumed to affect all substrates. In the prediction for rosuvastatin, we assume that the uptake rate of *1b is higher than that of *1a and *15. However, the observed *in vivo* *1b uptake rate is slightly lower than *1a rate.^21,22^ The predicted AUC_0–24 h_ of the *1b group (24.1 ng*·*h/ml) is less than the predicted AUC_0–24 h_ of the *1a group (32.6 ng*·*h/ml), in contrast with the observation that average AUC_0–*t*_ of *1b group is slightly higher than that of the *1a group.^21,22^ The reason for the inconsistency is unknown, but can be that protein expression differences do not proportionally translate to functional differences. Assuming that *1b does not increase OATP1B1 expression level in rosuvastatin studies, we re-estimate clearance for the three variants without the expression difference incorporated, leading to a result consistent with clinical observations (data not shown). In addition, if c.463 C>A is not sequenced in these study,^21,22^ subjects carrying *14 may exit in *1b or c.388A>G group. Since *14 may lead to reduction in intrinsic uptake rate as discussed above, it would compensate for increase in mean uptake rate due to *1b carriers.

The mechanistic modeling approach proposed here helps our understanding of the pharmacokinetic properties of OATP substrates in populations carrying OATP variants. For example, a previous *in vivo* study shows that the *SLCO1B1* polymorphism (i.e., SNP c.521T>C) has no impact on fluvastatin PK.^17^ Based on this result, several studies claim that fluvastatin is not an OATP1B1 substrate,^17,23,24^ which leads to an obvious inconsistency with *in vitro* uptake assay results where fluvastatin is an OATP1B1 substrate.^25^ This phenomenon can be explained using our approach. Previously published *in vitro* pharmacogenetic studies have shown that this SNP does not result in impaired OATP1B1 uptake activity for fluvastatin.^26,27^

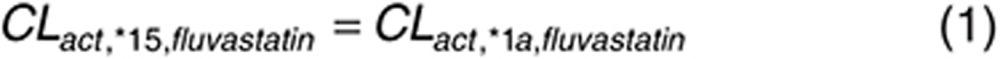


*CL*_*act, other, fluvastatin*_ is believed to be the same between *15 and *1a group here, hence

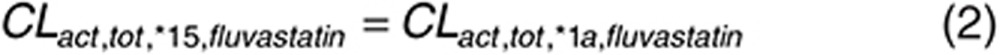


As such, even if fluvastatin is an OATP1B1 substrate as shown *in vitro*, SNP c.521T>C will not change the *in vivo* PK profile of fluvastatin, because *15 and *1a have the same hepatic clearance. In fact, the impact of *SLCO1B1* polymorphisms is known to be compound dependent.^27^ As such, *in vitro* functional evaluation of OATP polymorphisms can provide useful information on the prediction and interpretation of clinical pharmacokinetics.

In the sensitivity analysis, the rosuvastatin AUC_plasma_ is not sensitive to *CL*_act, *15_, mainly because given the current model parameter values, clearance of rosuvastatin by OATP1B1 *15 is not significant compared with clearance by other transporters. Similarly, pravastatin AUC_plasma_ is not sensitive to *CL*_*act, other*_, largely because the clearance of pravastatin by other transporter is not significant compared with clearance by OATP1B1. The genotyped *CL*_*act, tot*_ is calculated from *in vitro* data. To assess the impact of variability in the *in vitro* data on the calculated *CL*_*act, tot*_, we performed another sensitivity analysis (**Table 3**). Based on this analysis, if *in vitro* assay results indicate OATP1B1 mediates more than 60% of the *CL*_*act, tot*_, it is likely that the variability in the *in vitro* estimated intrinsic activity ratio between *CL*_act, *1a_ and *CL*_act, *1b_ will affect the calculation of the genotyped *CL*_*act, tot*_, and the PK prediction for each genotype. On the other hand, even if *CL*_*act, tot*_ is solely mediated by OATP1B1, the variability in the *in vitro* estimated ratio between *CL*_act, *1a_ and *CL*_act, *15_ may only affect *CL*_act, tot, *15_ estimation (results which are further illustrated in **Supplementary Figure S1**).

In this study, we performed analysis with PBPK rather than traditional pharmacokinetic modeling. For OATP substrates, the PBPK model has the ability to predict the pharmacokinetics in the liver, where tissue concentration to plasma concentration ratio is not constant. This is important in estimating efficacy for liver-targeting compounds (e.g., statins),^28,29^ potential liver toxicity of nonliver-targeting compounds (e.g., endothelin receptor antagonists),^30^ or potential drug–drug interactions in the liver.^31^ Additionally, although the current model uses nongenotyped human plasma data as a starting point, when combined with previously published modeling efforts to predict mean human pharmacokinetic response for compounds in the preclinical development,^11,12^ the model has the potential to prospectively predict pharmacokinetics in OATP1B1 genetic variant populations without using any human data.

Rose *et al.* recently published a study where a PBPK model was applied to assess the impact of OATP1B1 genetic variation on the pharmacodynamics of rosuvastatin.^29^ They estimated clearances by fitting genotyped human plasma data and evaluated the impact of OATP1B1 genetic variation on the pharmacodynamics. Our study uses *in vitro* estimated clearances together with nongenotyped clinical pharmacokinetics to prospectively predict how the OATP1B1 genetic variation affects the pharmacokinetics. Although using a different approach to evaluate the clearance values of the genotyped groups, and a slightly different liver model structure (three compartments (i.e., liver blood, liver extracellular tissue, and liver intracellular tissue) versus five pairs of liver blood and liver tissue) as well as different physiological parameters, the sensitivity analysis in our study reaches a similar conclusion as the published study:^29^ the rosuvastatin concentration in plasma is more sensitive to the genetic variability of OATP1B1 while the liver concentration is less sensitive. As such, the genetic variation in OATP1B1 may not affect the pharmacodynamic effects of liver-targeting compounds as much as the systemic pharmacokinetics. However, the OATP1B1 genetic variation may affect pharmacodynamics or toxicity in other tissues due to its effects on systemic exposure.

In conclusion, these results indicate that *in vitro* functional pharmacogenetic data can be used to support reasonably accurate predictions for groups carrying specific variants through the proposed PBPK modeling approach. Such an approach may be useful in the evaluation of drug candidates in drug discovery, the design of clinical trials and ultimately for dose adjustments in clinical practice. Lastly, this framework also provides a starting place from which to systematically evaluate some simplifying assumptions which are currently necessary due to the lack of information as more data become available (e.g., expression/activity of heterozygous vs. homozygous variants, expression/activity of variants beyond those examined in this particular study, gene interactions with other transporters).

## Methods

### PBPK modeling of nongenotyped clinical data and estimation of OATP1B1 activity

The structural model (**Supplementary Figure S2**) is based upon a previously published PBPK model.^12^ Equations were added to describe enterohepatic recirculation.

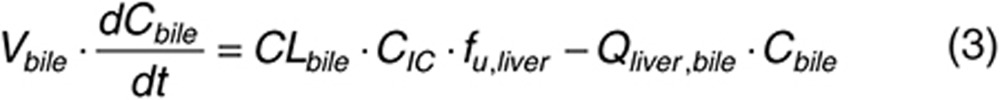


where *C*_*bile*_ and *C*_*IC*_ represent drug concentrations in the bile and liver tissue; *CL*_*bile*_ is biliary clearance; *f*_*u, liver*_ is the unbound fraction of compound in liver tissue; and *V*_*bile*_ and *Q*_*liver, bile*_ are the volume of bile ducts in the liver and the bile flow rate. *V*_*bile*_ and *Q*_*liver, bile*_ were 0.318% of liver volume^32^ and 350 ml/day.^33^ The GI lumen is modeled as

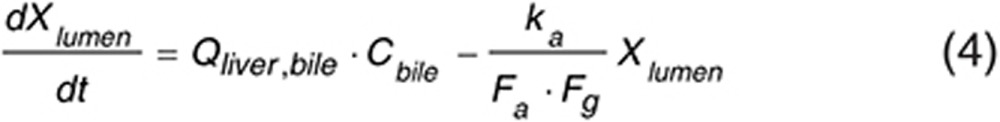


where *X*_*lumen*_ is the amount of compound in GI lumen compartment. *k*_*a*_ is the absorption rate; *F*_*a*_ is fractional absorption, *F*_*g*_ is the fraction that escapes from metabolism or efflux in the GI tract.

Gallbladder emptying after meals was not included in the modeling because feeding schedules were not available. The equation for the gut compartment is

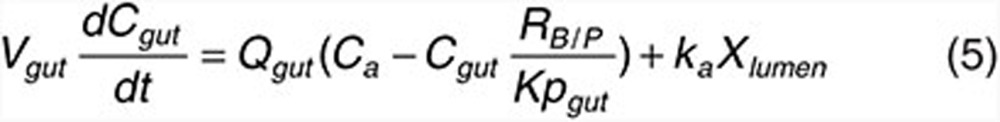


where *V*_*gut*_ is volume of gut; *Q*_*gut*_ is blood flow; *C*_*a*_ and *C*_*gut*_ are the concentrations in arterial blood and gut compartment; and *R*_*B/P*_ and *Kp*_*gut*_ are the blood to plasma ratio and tissue to plasma partition coefficient. (Equations and parameter values not reported in text are given in **Supplementary Material**.)

*CL*_*act, tot*_ is modeled as the sum of *CL*_*act, OATP1B1*_ and *CL*_*act, other*_:

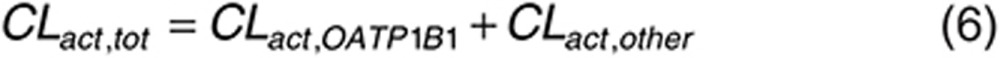


The nongenotyped *CL*_*act, tot*_, *CL*_*bile*_, *CL*_*pass*_, *k*_*a*_, *F*_*a*_*·F*_*g*_ were estimated by fitting the model to plasma concentration–time course data following intravenous infusion and oral dosing in nongenotyped studies.^6,7^ The ratios of *CL*_*pass*_ between liver, adipose and muscle and values of all other parameters are fixed at previously published values.^12^ The model is implemented in MATLAB (Version 2013a, Mathworks, Natick, MA) and differential equations are compiled as a MEX file (MATLAB code and MEX file are given in **Supplementary Material**). A stochastic global optimization method, differential evolution (DE) with nonlinear sampling, was used to estimate these parameters as described before.^34^ The 95% confidence intervals for the optimized parameters were approximated using a residual bootstrap method developed before.^12^

*F*_*a*_*·F*_*g*_ is also estimated using a previously published noncompartmental method^35^ to compare with the value estimated from fitting PBPK model to plasma data. The liver blood flow and *R*_*B/P*_ in the noncompartmental analysis are set to the values we used in the PBPK model.

### Predictions of uptake clearance in OATP variants and PK in genotyped Caucasian populations

To predict PK profiles of carriers of specific genetic variants, we keep all the parameters in PBPK model unchanged, but replace the nongenotyped *CL*_*act, OATP1B1*_ in Eq. 6 with predicted *CL*_act, *1a_, *CL*_act, *1b_, or *CL*_act, *15_ to generate new *CL*_*act, tot*_. For example, for the group carrying *15 after pravastatin dosing, we have

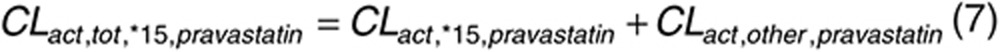


To simplify the problem, *5 is treated as *15 considering the relatively similar activities of the two variants.^8^ The diplotypes are restricted to homozygous *1a/*1a, *1b/*1b, and *15/*15 (or *5/*15, *5/*5). For the published concentration–time curves^15,16,17^ without information about SNP c.388A>G, we attribute the c.521TT group in these studies as *1a, and the c.521CC group as *5 and *15.

Consistent with that reported previously, we assumed the fraction of pravastatin uptake clearance due to OATP1B1 to be 83%.^23^ The remaining 17% is believed to be due to OATP1B3. We assume that pravastatin is not the substrate of other uptake transporter (e.g., Na^+^-taurocholate cotransporting polypeptide (NTCP)), because currently there is no evidence. However, if later research indicates other uptake transporter is involved in hepatic uptake of pravastatin, such information should be incorporated into calculation. With the fitted *CL*_*act, tot*_, we calculate *CL*_*act, OATP1B1*_ and *CL*_*act, other*_ for nongenotyped population as below:

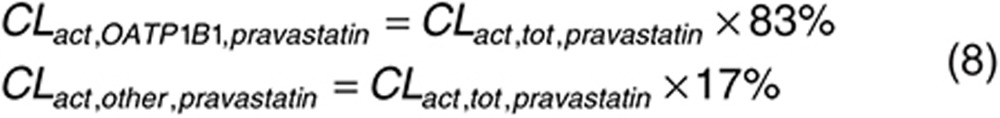


For rosuvastatin, 35% of total active uptake is due to NTCP activity,^36^ while 77% of the remaining 65% is mediated by OATP1B1.^37^ As such, OATP1B1 is expected to account for 50% of total uptake clearance of rosuvastatin.

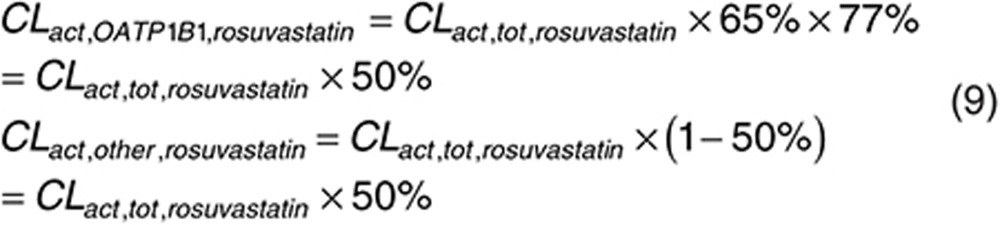


Because *CL*_*act, OATP1B1*_ is calculated from *CL*_*act, tot*_ estimated using mean data,^6,7^ *CL*_*act, OATP1B1*_ of nongenotyped population is treated as the average values of active uptake clearance of three major OATP1B1 genetic variants weighted by the proportion of participants carrying them (P_*1a_, P_*1b_, and P_*15_).

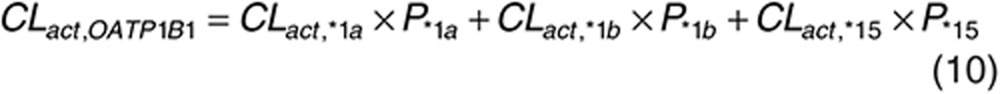


Since the participants were not genotyped for OATP1B1 polymorphisms in the published studies with intravenous infusion data from which we estimate *CL*_*act, tot*_,^6,7^ we assume that the proportion of OATP1B1 genetic variants follows the reported proportion in the European population (i.e., *1a, 56%; *1b, 26%; *5 and *15, 18%),^38^ considering the participants in the intravenous infusion studies were Caucasians.^6,7^

The ratios of intrinsic uptake clearances between *1a and *1b, and *1a and *15 are estimated from reported *in vitro* assay results. For pravastatin, the intrinsic uptake activities of *1b and *15 are reduced to 81% (ref. 8) and 35% (average value of the two reports)^8,26^ of the activity of *1a. The expression level of *1b is twice of *1a, while the expression level of *15 is about the same as *1a.^5^ Since a previous *in vivo* study has shown that *1b can lead to increased clearance,^20^ we assume that the active uptake clearance is proportional to the expression level.

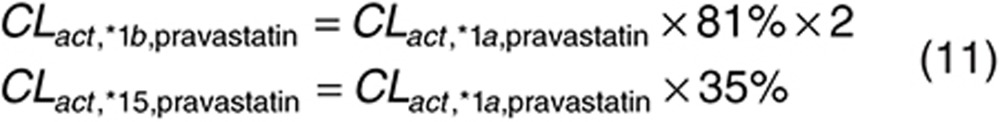


For rosuvastatin, similarly, the intrinsic uptake activities of *1b and *5&*15 are reduced to 82% (ref. 36) and 15% (average value of the two reports)^26,36^ of the activity of *1a. For prediction purpose, we assumed that *CL*_act,*1b_ for rosuvastatin is also increased due to the increased expression level, although this has not been supported by the clinical observation.^22^

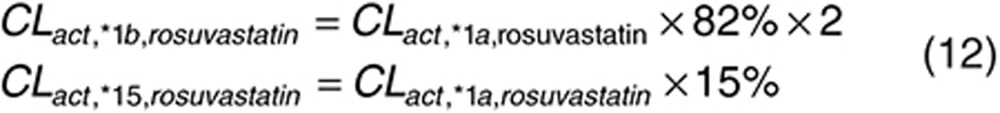


Combining Eqs. 10 with 11 and 12 we get what the expected mean clearance would be amongst the general population.

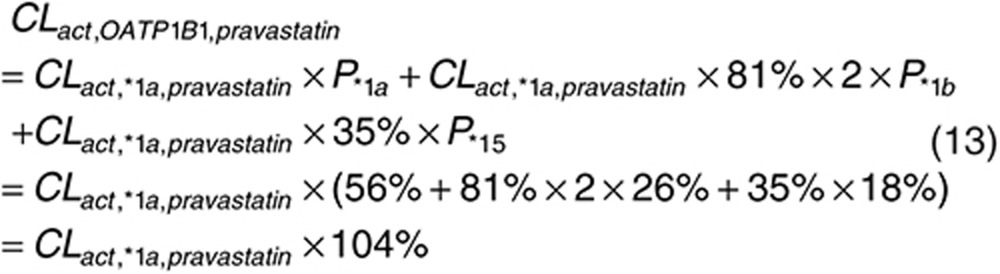


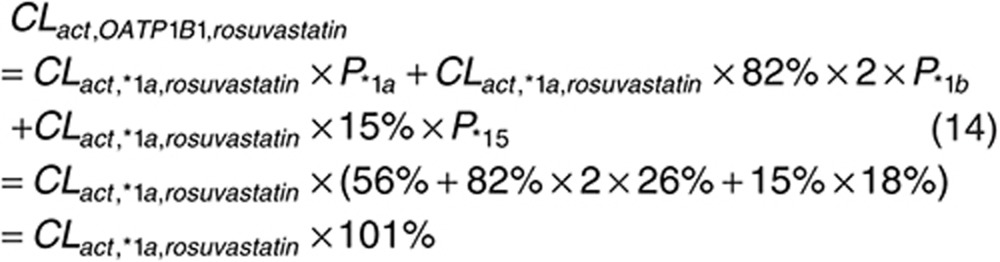


Finally, *CL*_act, *1a_, *CL*_act, *1b_, and *CL*_act, *15_ can be estimated from *CL*_*act, OATP1B1*_. With *CL*_*act, other*_ estimated above, *CL*_*act, tot*_ for three variants can be calculated using Eq. 7 and applied in PBPK model for PK predictions.

To compare predictions with observations, pravastatin human plasma concentration–time course data are digitized from four previously published studies, where Caucasian participants were genotyped for OATP1B1 polymorphisms and dosed orally with 40 mg pravastatin.^15,16,17,20^ Observed rosuvastatin human plasma concentration–time course data are also digitized from a previously published study, where Caucasian participants were genotyped for OATP1B1 polymorphisms and dosed orally with 10 mg rosuvastatin.^18^

### Predictions of uptake clearance in OATP variants and PK in genotyped Japanese populations

To test if the model can be applied to other populations by using a published ratio of intrinsic OATP1B1 activity between Japanese and Caucasians (i.e., 0.584),^9^ we correct *CL*_act, *1a_, *CL*_act, *1b_, and *CL*_act, *15_ with this value and assume that the ratio of Japanese/Caucasians for *CL*_*act, other*_ is also 0.584. Liver weight and hepatic blood flow are assumed to be same between Caucasian and Japanese populations.^39^ All other parameters are unchanged. To compare predictions with observations, clinical plasma concentration–time profiles are digitized from two published studies of pravastatin performed with Japanese populations.^13,14^ To date, similar data for rosuvastatin in a Japanese population are not available.

### Local sensitivity analyses

Local sensitivity analyses for the *in vivo* model were conducted as before^11^ where each compound specific parameter is raised by 1% with respect to its value in the PK simulations for genotyped Caucasian populations. The values of the plasma and liver concentrations throughout the time course, and AUC_plasma_ are obtained. Sensitivity coefficients are normalized to both the parameter value and the model output value.

In addition, using Eqs. 7–14, we evaluated the local sensitivity of predicted *CL*_*act, tot*_ for *1a, *1b, and *15 populations to the parameters estimated from the *in vitro* assays (i.e., intrinsic uptake activity ratio between *CL*_act, *1a_ and *CL*_act, *1b_; intrinsic uptake activity ratio between *CL*_act, *1a_ and *CL*_act, *15_; and the fraction of *CL*_*act, tot*_ mediated by OATP1B1). In this analysis, population parameters (i.e., P_*1a_, P_*1b_, P_*15_ for Caucasian population) and parameters estimated by fitting *in vivo* data (i.e., nongenotyped *CL*_*act, tot*_) are fixed. The intrinsic uptake activity ratio between *CL*_act, *1a_ and *CL*_act, *1b_ (or *CL*_act, *15_) is raised by 1%; and the value of the genotyped *CL*_*act, tot*_ (i.e., *CL*_act, tot, *1a_, *CL*_act, tot, *1b_, and *CL*_act, tot, *15_) is re-evaluated. The local sensitivity coefficient is calculated as the difference between the new value of the genotyped *CL*_*act, tot*_ and its nominal value (**Table 1**), divided by the nominal value and 1%. As the new value of genotyped *CL*_*act, tot*_ is also sensitive to the estimated fraction of *CL*_*act, tot*_ mediated (also determined *in vitro*), the sensitivity analysis was performed over a range of fractional OATP1B1 contributions between 0.2 and 1.

To visualize how actual interstudy variability in the *in vitro* data might impact these results, concentration–time profiles of pravastatin were generated over the range of *CL*_act, *1a_ and *CL*_act, *15_ intrinsic uptake activity ratios reported in the literature (i.e., 0.20 (ref. 8) and 0.50 (ref. 26)). The ratio between *CL*_act, *1a_ and *CL*_act, *15_ of pravastatin is selected because (i) its value is available from two independent studies, while values of most other parameters are only available from single *in vitro* studies; (ii) its value of pravastatin shows the largest difference between two studies (the value for rosuvastatin is also available from two studies, which however reported almost identical numbers);^26,36^ and (iii) OATP1B1 contributes over 80% of total hepatic active uptake of pravastatin, hence has a more pronounced impact on pharmacokinetics. The intrastudy variability is not included in the current study, considering it is generally small with the coefficient of variation below 60%.^5,8,26,36,37^

## Author contributions

T.M., R.L., and H.B. wrote the manuscript. T.M., R.L., and H.B. designed the research. R.L. performed the research. R.L. analyzed the data

## Conflict of interest

The authors declared no conflict of interest.

## Study Highlights


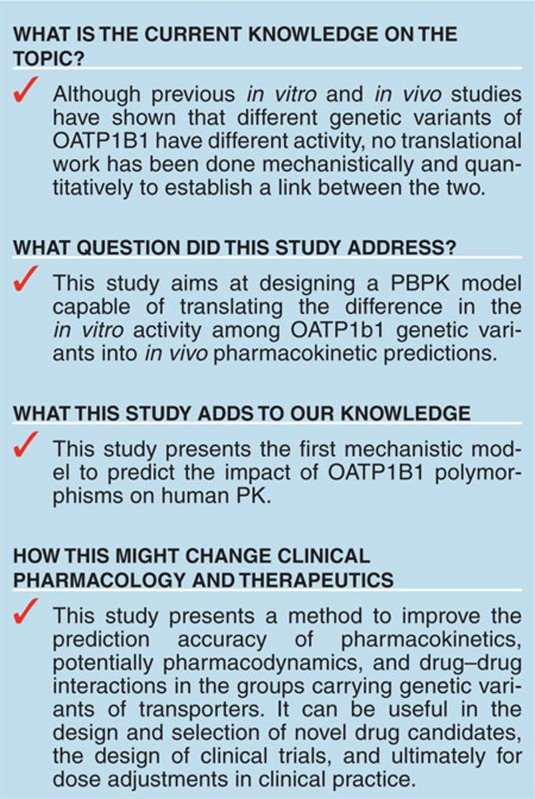


## Figures and Tables

**Table 1:**
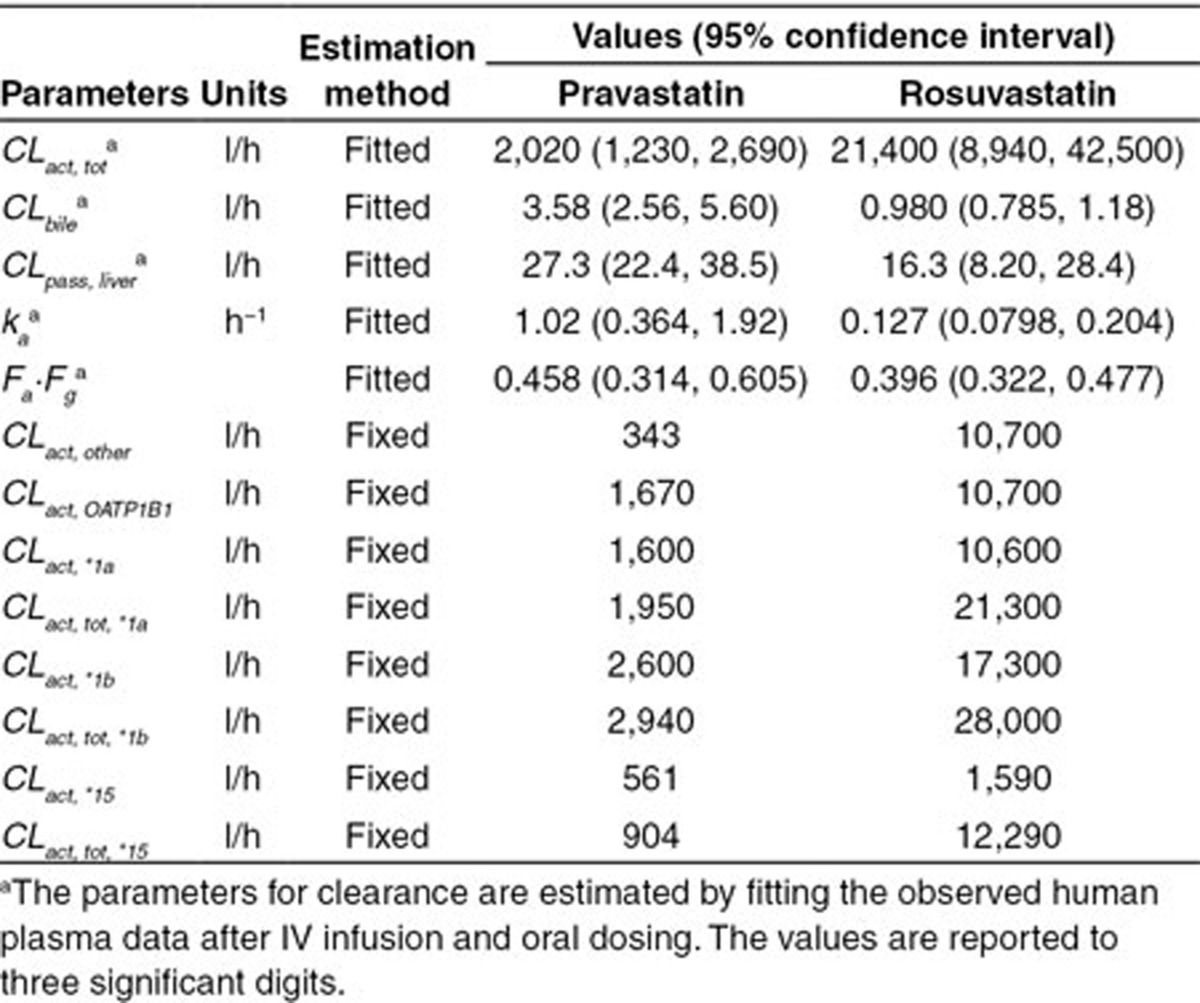
Values of parameters for clearance and absorption

**Table 2:**
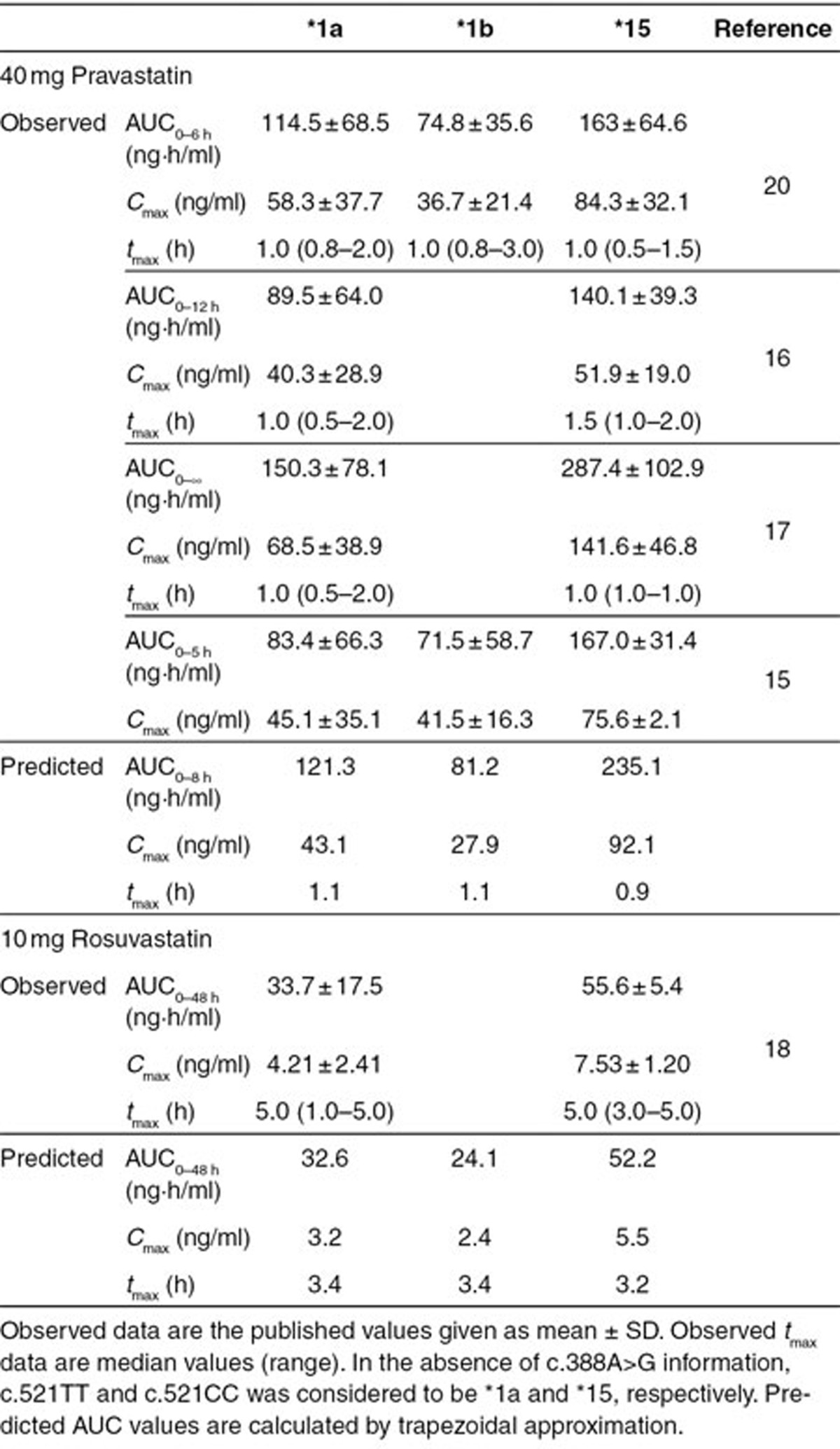
Observed and predicted pharmacokinetic variables in relation to OATP1B1 polymorphism

**Table 3:**
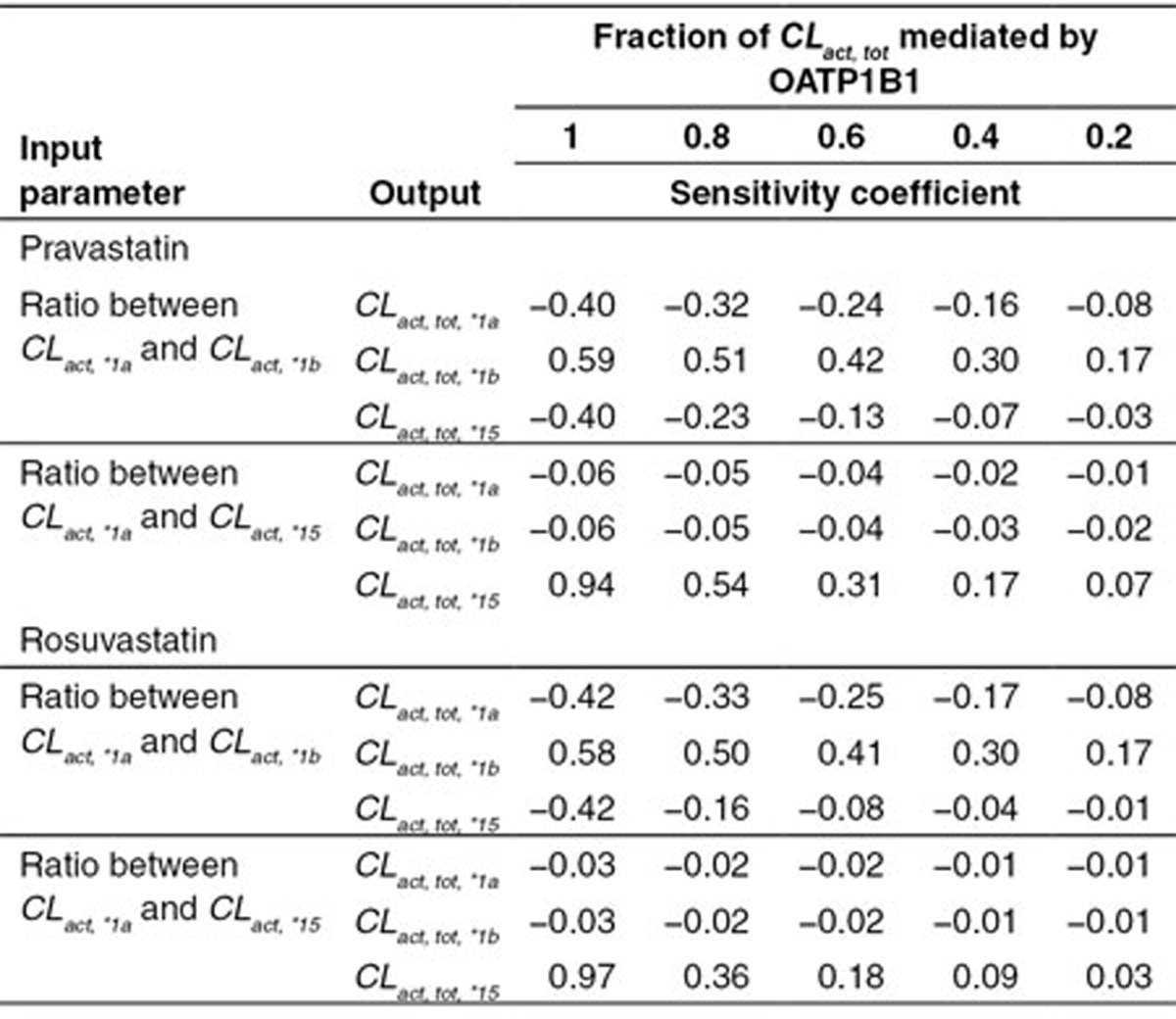
The local sensitivity analysis on *CL*_*act, tot*_ of different genetic variant groups with respect to the parameters estimated in the *in vitro* assays
