# Supplementary material for: Toward Prospective Prediction of Pharmacokinetics in OATP1B1 Genetic Variant Populations
Source: CPT Pharmacometrics Syst Pharmacol. 2014 Dec 10;3(12):e151–. doi: 10.1038/psp.2014.50 (PMC4288003; doi:10.1038/psp.2014.50)
Supplement: Supplementary Information [file psp201450x1.zip › PSP-2014-0056-s03.docx]

**Supplementary Materials**

**Mathematical modeling**

All compartments were connected by the circulating blood system. Compound distribution in tissues, except liver, adipose and muscle, was assumed to be perfusion limited by tissue-plasma partition coefficient (*Kp*) according to the relationship

$$V_{T}\frac{dC_{T}}{dt}=Q_{T}\cdot C_{a}-Q_{T}\cdot C_{T}\cdot\frac{R_{B/P}}{Kp_{T}},$$

where *Q* is blood flow (L/hr), *C* is concentration (µg/L), and *V* is volume. *T* and *a* represent tissue and artery, respectively. *Kp* values were estimated using standard *in silico* method (1). For the compounds with renal CL, we used the unbound renal intrinsic clearance (*CL_renal_*) calculated from the reported values, assuming well-stirred conditions (2). For kidney we have

$$V_{kidney}\frac{dC_{kidney}}{dt}=Q_{kidney}\cdot C_{a}-Q_{kidney}\cdot C_{kidney}\cdot\frac{R_{B/P}}{Kp_{kidney}}-\text{CL}_{\text{renal}}\cdot C_{kidney}\cdot\frac{f_{u,p}}{Kp_{kidney}},$$

Adipose and muscle were modeled with 2 sub-compartments (extracellular (EC) and intracellular spaces (IC)), where compound distribution is limited by passive diffusion clearance (*CL_u, pass, T_*) of unbound drugs.

The permeability between tissue EC and IC is assumed to be the same as the permeability between liver EC and IC, hence the ratio of passive intrinsic clearance (*CL_int, u, pass_*) between a hepatocyte (H) and a tissue cell (T) is assumed to be the ratio of their surface areas (SA).

$$CL_{pass,T}=CL_{pass,liver}\cdot\frac{SA_{T}}{SA_{H}}.$$

The liver was modeled as a permeability-limited tissue with 5 sequential pairs of 2 sub-compartments.

For the first EC compartment

$$\begin{matrix} V_{EC,1}\frac{dC_{EC,1}}{dt}=Q_{ha}\cdot C_{a}+Q_{gut}\cdot C_{gut}\cdot\frac{R_{B/P}}{Kp_{gut}}+Q_{spleen}\cdot C_{spleen}\cdot\frac{R_{B/P}}{Kp_{spleen}}-Q_{liver}\cdot C_{EC,1} \\ -\frac{\text{CL}_{\text{u,pass}}}{5}\cdot\left[C_{EC,1}\cdot\frac{f_{u,p}}{R_{B/P}}-C_{IC,1}\cdot f_{u,liver}\right]-\frac{\text{CL}_{\text{u,act}}}{5}\cdot C_{EC,1}\cdot\frac{f_{u,p}}{R_{B/P}}, \end{matrix}$$

For the *i* = 2 - 5 compartments

$$\begin{matrix} V_{EC,i}\frac{dC_{EC,i}}{dt}=Q_{liver}\cdot\left[C_{EC,i-1}-C_{EC,i}\right]-\frac{\text{CL}_{\text{u,pass}}}{5}\cdot\left[C_{EC,i}\cdot\frac{f_{u,p}}{R_{B/P}}-C_{IC,i}\cdot f_{u,liver}\right] \\ -\frac{\text{CL}_{\text{u,act}}}{5}\cdot C{}_{EC,i}\cdot\frac{f_{u,p}}{R_{B/P}}, \end{matrix}$$

For the first IC compartment

$$\begin{matrix} V_{IC,1}\frac{dC_{IC,1}}{dt}=\frac{CL_{pass,liver}}{5}\cdot\left[C_{EC,1}\cdot\frac{f_{u,p}}{R_{B/P}}-C_{IC,1}\cdot f_{u,liver}\right]-\frac{CL_{pass,liver}}{5}\cdot\left[C_{IC,1}-C_{IC,2}\right]\cdot f_{u,liver} \\ +\frac{CL_{act,tot}}{5}\cdot C{}_{EC,1}\cdot\frac{f_{u,p}}{R_{B/P}}-\frac{CL_{bile}}{5}\cdot C{}_{IC,1}\cdot f_{u,liver}. \end{matrix}$$

For the *i* = 2 - 4 compartments

$$\begin{matrix} V_{IC,i}\frac{dC_{IC,i}}{dt}=\frac{CL_{pass,liver}}{5}\cdot\left[C_{EC,i}\cdot\frac{f_{u,p}}{R_{B/P}}-C_{IC,i}\cdot f_{u,liver}\right]-\frac{CL_{pass,liver}}{5}\cdot\left[C_{IC,i}-C_{IC,i+1}\right]\cdot f_{u,liver} \\ +\frac{CL_{pass,liver}}{5}\cdot\left[C_{IC,i-1}-C_{IC,i}\right]\cdot f_{u,liver}+\frac{CL_{act,tot}}{5}\cdot C{}_{EC,i}\cdot\frac{f_{u,p}}{R_{B/P}}-\frac{CL_{bile}}{5}\cdot C{}_{IC,i}\cdot f_{u,liver}. \end{matrix}$$

For the fifth compartment,

$$\begin{matrix} V_{IC,5}\frac{dC_{IC,5}}{dt}=\frac{CL_{pass,liver}}{5}\cdot\left[C_{EC,5}\cdot\frac{f_{u,p}}{R_{B/P}}-C_{IC,5}\cdot f_{u,liver}\right]+\frac{CL_{pass,liver}}{5}\cdot\left[C_{IC,4}-C_{IC,5}\right]\cdot f_{u,liver} \\ +\frac{CL_{act,tot}}{5}\cdot C{}_{EC,5}\cdot\frac{f_{u,p}}{R_{B/P}}-\frac{CL_{bile}}{5}\cdot C{}_{IC,5}\cdot f_{u,liver}. \end{matrix}$$

All the compound specific parameters that are not listed in Table 1 are given in Table S1. Equations for enterohepatic recirculation are given in the text. The species-dependent parameter values used in this study have been published before (3). The fraction of intracellular and extracellular tissue volumes in the liver, adipose, and muscle are published by (4, 5). All the parameter values are also available in the MATLAB files (pbpk_rosuvastatin_CL_Global.m, pbpk_rosuvastatin_CL_PGx.m, pbpk_pravastatin_CL_Global.m, and pbpk_pravastatin_CL_PGx.m).

**MATLAB codes**

Short descriptions of MATLAB codes in the Supplementary Materials are listed here. Other than MEX files, the MATLAB scripts can be opened and edited.

**pbpk_EC_permeableT_permeableLiver.mexw64:** MATLAB MEX file for solving differential equations (64 bit Windows OS)

**pbpk_EC_permeableT_permeableLiver.mexw32:** MATLAB MEX file for solving differential equations (32 bit Windows OS)

**pbpk_rosuvastatin_CL_Global.m:** The MATLAB code passing all parameters values of rosuvastatin to equation solver (non-genotyped)

**pbpk_pravastatin_CL_Global.m:** The MATLAB code passing all parameters values of pravastatin to equation solver (non-genotyped)

**Fitting_dev_CL_Global.m:** The MATLAB code calculating objective function value (non-genotyped)

**simulation_CL_Global.m:** The MATLAB code plotting simulated and observed plasma concentration time profiles (non-genotyped)

**pbpk_rosuvastatin_CL_PGx.m:** The MATLAB code passing all parameters values of rosuvastatin to equation solver (OATP1B1-genotyped)

**pbpk_pravastatin_CL_PGx.m:** The MATLAB code passing all parameters values of pravastatin to equation solver (OATP1B1-genotyped)

**simulation_PGx.m:** The MATLAB code plotting simulated and observed plasma concentration time profiles (OATP1B1-genotyped)

In this study, “devec3_nus.m”, a differential evolution (DE) optimizer with non-uniform sampling implemented in MATLAB, was use to optimize parameter values. “devec3_nus.m” code was developed by Kleinstein, S. H. et al. ([steven.kleinstein@yale.edu](mailto:steven.kleinstein@yale.edu)) based on DE code (“devec3.m”, <http://www.icsi.berkeley.edu/~storn/code.html> ). The “devec3_nus.m” code is located at <http://www.cs.princeton.edu/~stevenk/optimization/>

To optimize parameters in “Fitting_dev_CL_Global_pravastatin.m” offered in Supplementary Materials, following commands should be executed in MATLAB:

| ub = [1e5,1e3,1e2,1e1,1];  lb = [10,1e-1,1e-2,1e-3,1e-4];  [Phi,OFV,~] = devec3_nus('Fitting_dev_CL_Global_pravastatin', ...  -Inf,5,lb,ub,[],20*5,200,0.5,0.8,3,10) |
| --- |

where ub and lb are upper and lower bounds of parameters, Phi is a vector of optimized parameter (i.e. *CL_act, tot_*, *CL_pass, liver_*, *CL_bile_*, *k_a_*, *F_a_F_g_*) values, and OFV is final objective function value. Details about options and inputs for “devec3_nus.m” are given at the beginning of the code. To plot simulated and observed plasma concentration time profiles in non-genotyped population, following commands should be executed in MATLAB:

| OFV = simulation_CL_Global_pravastatin(Phi); |
| --- |

where Phi is the vector of parameter (i.e. *CL_act, tot_*, *CL_pass, liver_*, *CL_bile_*, *k_a_*, *F_a_F_g_*) values. To plot simulated and observed plasma concentration time profiles in OATP1B1-genotyped population, following commands should be executed in MATLAB:

| OFV = simulation_PGx_pravastatin; |
| --- |

**supplementary table**

Table S1 compound-specific parameter values

| Parameter | Value | | Reference |
| --- | --- | --- | --- |
|  | Pravastatin | Rosuvastatin |  |
| *logD_7.4_* | −0.84 | −0.33 | (2) |
| *pKa* | 4.6 | 4.2 | (2) |
| *f_u, p_* | 0.42 | 0.094 | (2) |
| *f_u, liver_* | 0.45 | 0.60 | (5) |
| *R_B/P_* | 0.55 | 0.56 | (2) |
| *CL_renal_* (L/hour) | 174 | 186 | (2) |
| *CL_pass, adipose_* (L/hour) | 82.6 | 49.3 | This study |
| *CL_pass, muscle_* (L/hour) | 7060 | 4220 | This study |
| *Kp_adipose_* | 0.09 | 0.06 | (1) |
| *Kp_bone_* | 0.16 | 0.11 | (1) |
| *Kp_brain_* | 0.20 | 0.08 | (1) |
| *Kp_gut_* | 0.30 | 0.19 | (1) |
| *Kp_heart_* | 0.27 | 0.18 | (1) |
| *Kp_kidney_* | 0.26 | 0.16 | (1) |
| *Kp_lung_* | 0.31 | 0.23 | (1) |
| *Kp_muscle_* | 0.19 | 0.09 | (1) |
| *Kp_skin_* | 0.38 | 0.30 | (1) |
| *Kp_spleen_* | 0.21 | 0.12 | (1) |
| *Kp_testes_* | 0.19 | 0.09 | (1) |
| *Kp_rest body_* | 0.19 | 0.09 | (1) |

**Supplementary Figure Legends**

Figure S1. Simulated human plasma time-concentration profiles of pravastatin after 40 mg oral dosing in (A) *1a, (B) *1b, and (C) *15 groups, with changes in the ratio between *CL_act, *1a_* and *CL_act, *15_*. The red dashed, blue dot-dashed, and black solid lines represent simulations with the value of this ratio of 0.20 (6), 0.50 (7), and 0.35 (mean of the two reported values).

Figure S2. Schematic diagram of the *in vivo* PBPK model. EC, extracellular; IC, intracellular. Solid arrows, blood flow; dashed arrow, absorption; dotted arrows, clearances; (1), passive diffusion clearance; (2), active uptake clearance; (3), metabolic clearance; (4), biliary clearance; (5), renal clearance.

**Supplementary reference**

(1) Rodgers, T. & Rowland, M. Physiologically based pharmacokinetic modelling 2: predicting the tissue distribution of acids, very weak bases, neutrals and zwitterions. *J Pharm Sci* **95**, 1238-57 (2006).

(2) Jones, H.M. *et al.* Mechanistic pharmacokinetic modeling for the prediction of transporter-mediated disposition in humans from sandwich culture human hepatocyte data. *Drug metabolism and disposition: the biological fate of chemicals* **40**, 1007-17 (2012).

(3) Jones, H.M., Parrott, N., Jorga, K. & Lave, T. A novel strategy for physiologically based predictions of human pharmacokinetics. *Clinical pharmacokinetics* **45**, 511-42 (2006).

(4) Watanabe, T., Kusuhara, H., Maeda, K., Shitara, Y. & Sugiyama, Y. Physiologically based pharmacokinetic modeling to predict transporter-mediated clearance and distribution of pravastatin in humans. *The Journal of pharmacology and experimental therapeutics* **328**, 652-62 (2009).

(5) Li, R. *et al.* A "middle-out" approach to human pharmacokinetic predictions for OATP substrates using physiologically-based pharmacokinetic modeling. *Journal of pharmacokinetics and pharmacodynamics* **41**, 197-209 (2014).

(6) Kameyama, Y., Yamashita, K., Kobayashi, K., Hosokawa, M. & Chiba, K. Functional characterization of SLCO1B1 (OATP-C) variants, SLCO1B1*5, SLCO1B1*15 and SLCO1B1*15+C1007G, by using transient expression systems of HeLa and HEK293 cells. *Pharmacogenetics and genomics* **15**, 513-22 (2005).

(7) Choi, M.K., Shin, H.J., Choi, Y.L., Deng, J.W., Shin, J.G. & Song, I.S. Differential effect of genetic variants of Na(+)-taurocholate co-transporting polypeptide (NTCP) and organic anion-transporting polypeptide 1B1 (OATP1B1) on the uptake of HMG-CoA reductase inhibitors. *Xenobiotica; the fate of foreign compounds in biological systems* **41**, 24-34 (2011).
