# Supplementary figures and images for: Toward Prospective Prediction of Pharmacokinetics in OATP1B1 Genetic Variant Populations
Source: CPT Pharmacometrics Syst Pharmacol. 2014 Dec 10;3(12):e151–. doi: 10.1038/psp.2014.50 (PMC4288003; doi:10.1038/psp.2014.50)

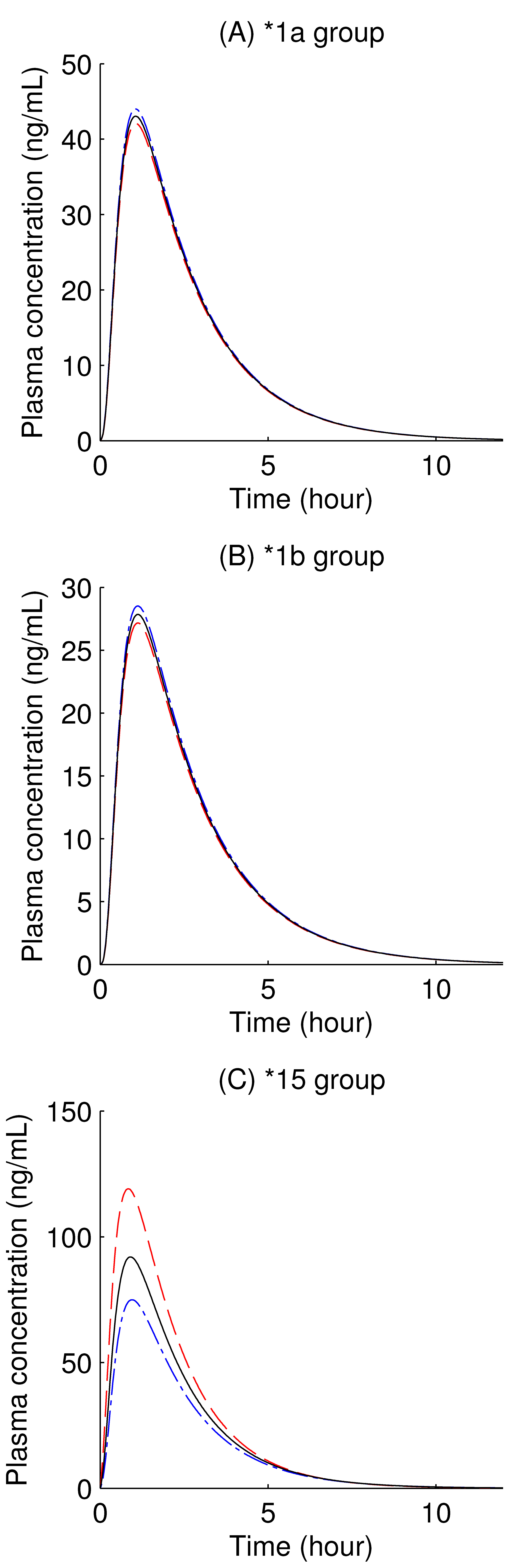

Supplement: Supplementary Information [file psp201450x1.zip › PSP-2014-0056-s04.jpg]

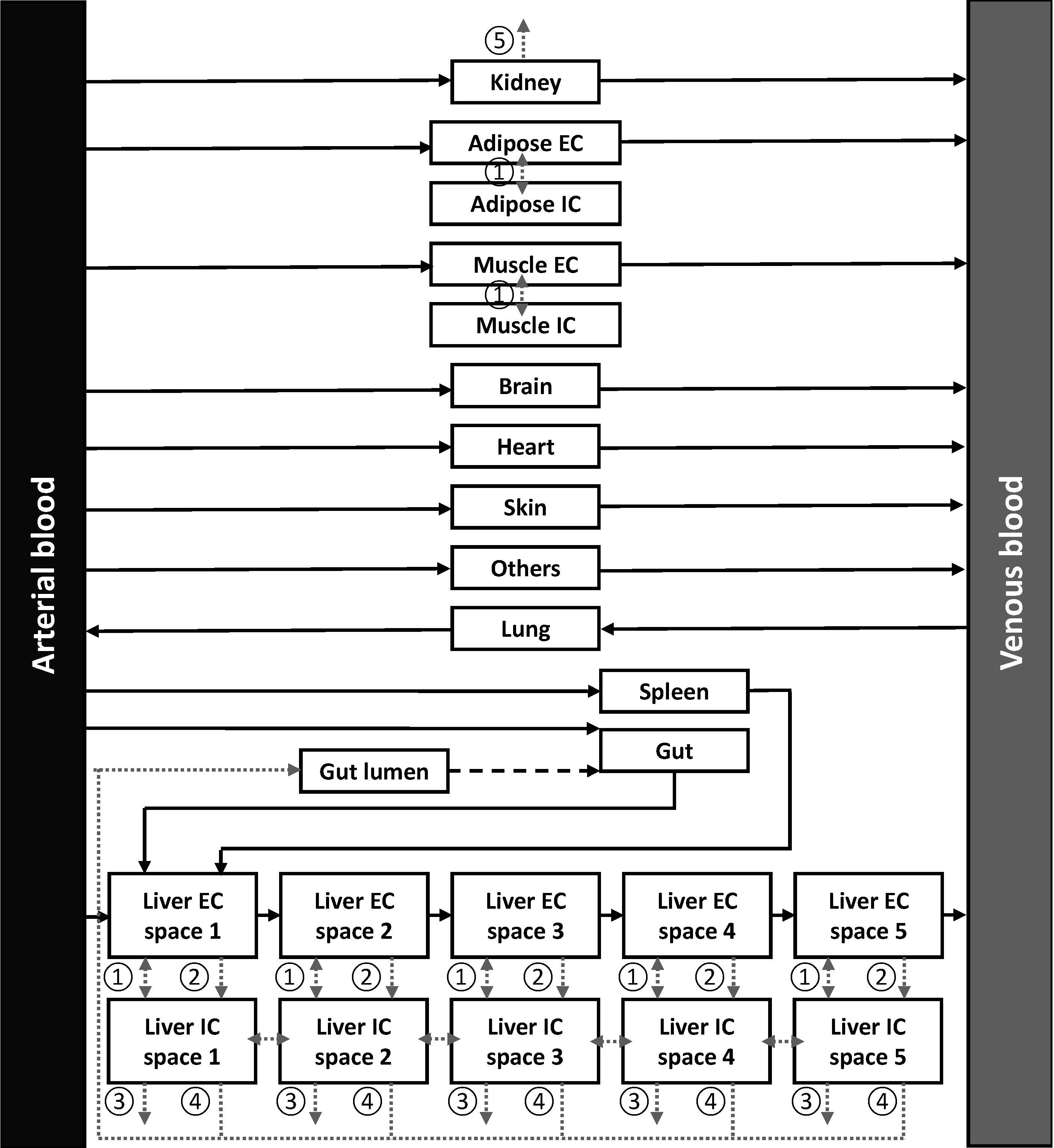

Supplement: Supplementary Information [file psp201450x1.zip › PSP-2014-0056-s05.jpg]
